# Amperometric Biosensors for Real Time Assays of Organophosphates

**DOI:** 10.3390/s8095303

**Published:** 2008-09-01

**Authors:** Miroslav Pohanka, Daniel Jun, Kamil Kuca

**Affiliations:** 1 Center of Advanced Studies, Faculty of Military Health Sciences, University of Defence, Hradec Kralove, Czech Republic; E-mails: jun@pmfhk.cz; kucakam@pmfhk.cz; 2 Department of Toxicology, Faculty of Military Health Sciences, University of Defence, Hradec Kralove, Czech Republic; 3 Center of Biological Defense, Techonin, Czech Republic

**Keywords:** Organophosphate, paraoxon, electrochemical, biosensor, acetylcholinesterase, assay

## Abstract

An amperometric biosensor based on acetylcholinesterase (AChE) immobilized in gelatin was used to develop an assay for the organophosphate paraoxon. The more traditional manner employing preincubation was used for comparison between measurement procedures, although the aim of the study was to examine the performance of the biosensor for real time monitoring of organophosphates. The biosensor was immersed in a reaction chamber and paraoxon was injected inside. We were able to detect 200 pg of paraoxon within one minute or 2.5 ppb when the biosensor was preincubed in the sample solution for 15 minutes. The practical impact and expectations are discussed.

## 1. Introduction

The organophosphates constitute a group of important compounds found both in Nature as well as man-made preparations. The well known man-made organophosphates are pesticides used worldwide in agriculture and nerve agents misused for military and terrorist purposes. Diazinon, dichlorvos, malathion, parathion, and paraoxon are probably the most common organophosphorus pesticides. These organophosphorus pesticides generally compare quite favorably with the organochlorine pesticides due to their spontaneous hydrolysis in the environment, resulting in less residual activity. Sarin, tabun, soman and VX are representatives of the nerve agent group. A common effect of organophosphorus pesticides and nerve agents is the inhibition of acetylcholinesterase (AChE; EC 3.1.1.7) and butyrylcholinesterase (BChE; EC 3.1.1.8) by way of catalytically active serine phosphorylation [1]. Although binding of organophosphate to the active centre is covalent, reactivation seems to be possible. Reactivation of nerve agent inhibited AChE was extensively investigated [2, 3]. In another study, the reactivation of paraoxon-inhibited AChE by obidoxime, pralidoxime, and HI-6 was described [4].

The above described cholinesterase inhibition is commonly considered a negative process. On the other hand, analytical procedures based on monitoring of cholinesterase activity are emerging. Some discrepancies can occur when heavy metals are present in the samples but typically cholinesterases can be measured favourably [5]. An AChE based assay in the form of simple dipstick can be performed. Thus, No *et al.* developed an AChE coated membrane strip for organophosphate and carbamate pesticide assays [6]. In another study, Kim *et al.* presented an AchE-based kit for monitoring of pesticides present in agricultural samples [7]. Cholinesterase immobilization on any suitable physicochemical transducer leads to the formation of biosensor. Some strategies for biosensor construction were described by Skladal [8]. Trends in cholinesterase-based biosensors were summarized and these biosensors were compared with the one based on organophosphorus hydrolase [9]; although organophosphorus hydrolase based biosensors were referred to as intriguing, cholinesterase based biosensors were found more convenient for low detection limit assays. AChE can be combined with another enzyme – cholineoxidase, thus forming choline which is oxidized into betaine and the secondary reaction product hydrogen peroxide is measured amperometrically [10, 11]. However, the more common biosensor design is based on immobilization of only AChE and acetylthiocholine as substrate in combination with an amperometric transducer. The reaction product thiocholine is oxidized at the working electrode. This mode of analysis was used by researchers in several studies [12-14]. This type of bisensor was also adapted for a pharmacology prescreening study of reactivator HI-6 competition [15]. The applied potential needed for thiocholine oxidation can be decreased in the presence of hexacyanoferrate [16] or Prussian blue [17]. Another way to improve biosensors is through the use of nanostructured electrode materials [18]. The activity of cholinesterases can be followed by a potentiometric electrode system too [19]. The main disadvantage of potentiometric biosensors is the inability to perform assays in strongly buffered solutions.

Although biosensors with AChE and/or BChE as biorecognition elements evolved many years ago, continuous improvements in physicochemical transducers and the quality of commercially available cholinesterases has led to a continuous improvements in biosensor performance [20]. In the work presented herein, we continue our efforts to develop devices for fast detection and monitoring of organophosphates. Typical systems based on AChE typically need some preincubation time, but we have developed a biosensor for fast analysis that is critical for anticipation of organophosphate misuse.

## 2. Results and Discussion

### 2.1. Biosensor preparation

Four basic AChE immobilization procedures were tested: simple sorption on a platinum working electrode, sorption combined with cross-linking by glutaraldehyde, cross-linking by glutaraldehyde with human serum albumin (HSA) and capture in a gelatin membrane. The prepared biosensors were characterized according to the maximum current provided by the non-inhibited biosensor (*i_n_*). The biosensors were inhibited by 1,000 ppb paraoxon for one hour and the current *i_i_* was determined. Percent of inhibition (*I*) was then computed. Results are presented in Table 1:

A significantly higher current was provided by the biosensor with AChE captured in gelatin. Other immobilization procedures were less efficient. The gelatin based AChE immobilization was more effective than the previously described one based on glutaraldehyde cross-linking [21]. On the other hand, the percent of inhibition covered a narrow range. Nevertheless, the procedure based on capture on gelatin was the best. Gelatin is a quite cheap reagent. Furthermore, the reproducibility of this immobilization was high so we consider immobilization based on AChE capture on gelatin as the most optimal one. Subsequent experiments were performed using only this immobilization procedure.

### 2.2. Measuring procedure employing a preincubation step

**Figure 1.**
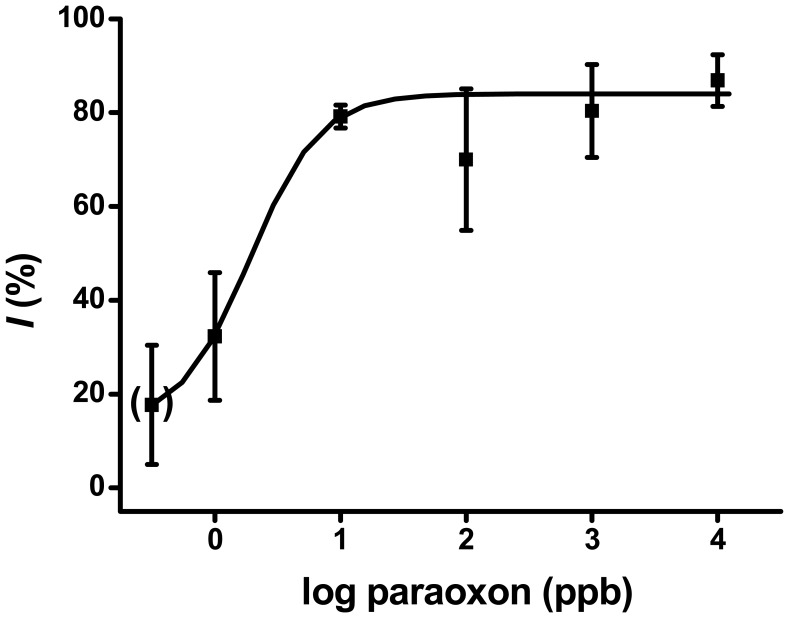
Calibration curve for paraoxon. Percentage of inhibition (*I*) vs. logarithm of paraoxon concentration (ppb) is presented. The error bars indicates standard deviation (n=3, where n is independent assay by a new biosensor).

The usual mode of application of biosensors for AchE-based recognition requires a long preincubation period, after which it is possible to detect very low levels of organophosphate, with a limit of detection as low as 2.5 ppb of paraoxon in water solutions. The main disadvantage is the obvious fact that this method is very time consuming. Our preincubation takes fifteen minutes; approximately 5 minutes are needed for *i_n_*, *i_i_* measurement and biosensor manipulation. The calibration curve for paraoxon concentrations 1 – 10,000 ppb is presented in Figure 1.

Paraoxon concentrations above 10,000 ppb did not provide higher inhibition percentages. Inhibition seems to be limited to 90%. The potential influence of the background signal on the detected percent inhibition was also tested. Sensors with only gelatin (without AChE) and immersed into the reaction cell provided steady state currents of less than 2 nA, typically 0.5 nA. This value is approximately eighty times lower than the one provided by a biosensor with AChE and was on the edge of the amperometric detector detection limit, so we conclude that the contribution of the background current to the finally detected inhibition was insignificant. Another convenient way of improving the detection limit is by sample preincubation. We extend the preincubation extension up to forty minutes. Although slightly higher inhibitions occured, the improvements was disproportionally lower than the additonal time required.

### 2.3. Real time monitoring of organophosphates presence

Monitoring of organophosphates by AChE based biosensors in real time is not a typical procedure. Our study is aimed in achieving this. We anticipate a practical impact when the problem of real time monitoring of organophosphates can be solved. The biosensor was placed within the reaction chamber during the whole measuring setup. Only 2 μL of organophosphate were injected into the chamber. This amount approximately corresponds to the volume of aerosol particles so we expect that further studies will allow detection of aerosol particles with organophosphates. A real time record of an analysis is illustrated below.

The real time curves demonstrate the feasibility of the biosensor. The higher concentration of paraoxon caused rapid inhibition. E.g. when the concentration of paraoxon exceeds 100 ppb, the half time of inhibition was less than 40 seconds. This value was obtained using the real record data, fitted by exponential decay using the Origin software. Indeed, the much lower concentration of paraoxon causes an extension of the half time of inhibition due to limited diffusion distribution of paraoxon on the biosensor surface.

**Figure 2.**
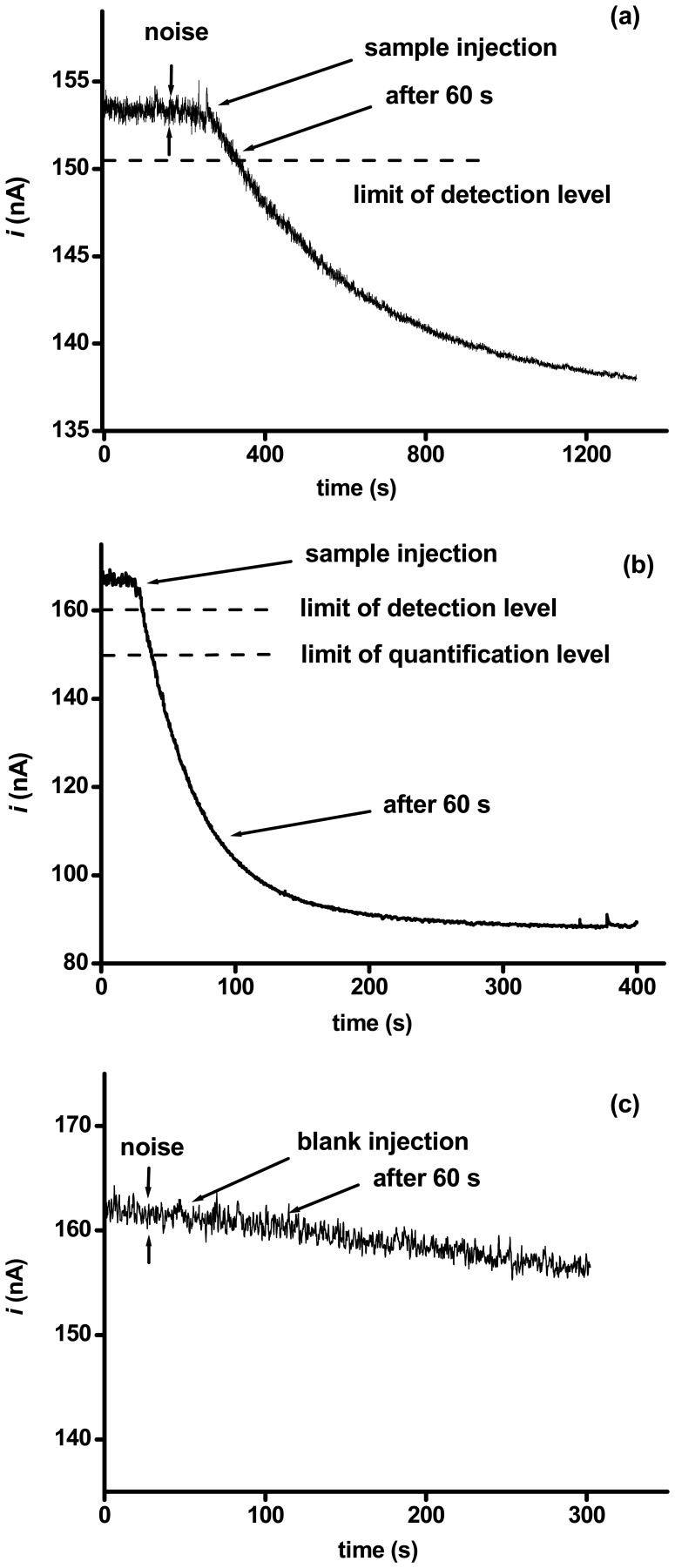
The curves represent following of AChE based biosensor inhibition by organophosphate paraoxon. The graph marked by letter *a* was obtained by injection 20 pg of paraoxon; the one marked by letter *b* is meaning for injection 2 ng of paraoxon. The most important moments of analysis (limit of detection and quantification leves, noise, moment of sample injection and time moment equal to 60 s after sample injection) are indicated in graphs by arrows and lines The graph marked by letter *c* represents blank application, drift of background signal could be observed in this graph.

**Figure 3.**
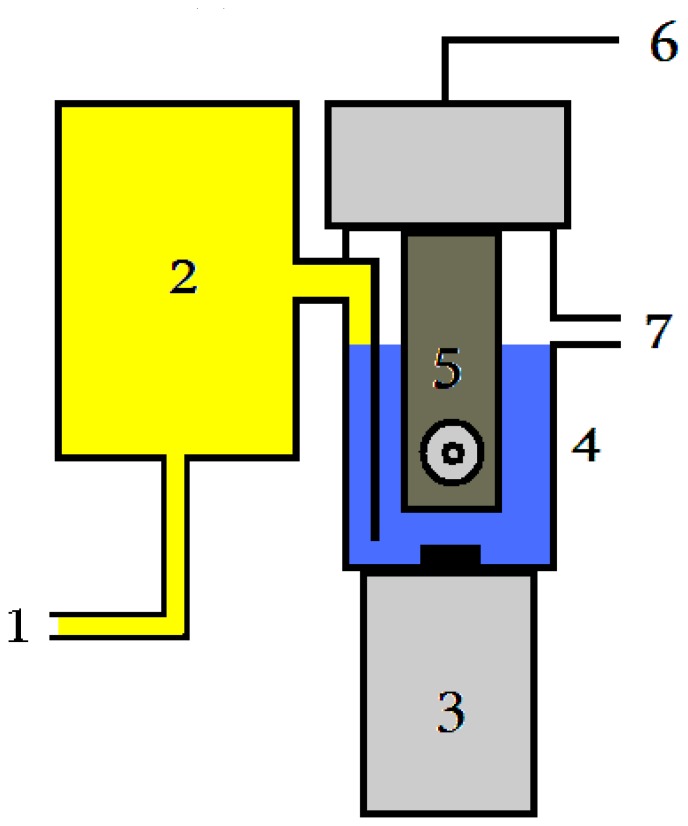
Prospective concept of device for detection of organophosate pesticides and nerve agents. The main parts are followings: input of sample (1); pumping of sample eventually mixing with a fresh substrate (2); magnetic stirring (3); reaction cell (4); biosensor based on screen printed electrodes and immobilized AChE (5); signal output (6); outlet of superfluous substrate (7).

The main aim of this study was the development of devices for monitoring for the presence of organophosphates. To this end we do not consider a precise inhibition value as critical and we rather prefer a simple evaluation scale. According to the experimental section, we elected simple scale consisting of “−, +, ++”. The limit of detection is strongly dependent on the recording time. E.g. graph *a* in Figure 2 represents a measurement of organophosphate at a concentration below the limit of detection when we consider one minute as the critical time moment. Paraoxon could be positively detected a short time after this moment. The real records were compared with the ones obtained by injection of deionized water or PBS (data not shown). Although a slight current shift was observed, the shift was under the level of the limit of detection and current finally fluctuated around the initial value. We have to explain why one minute was selected as a critical moment. This time is commonly considered as an optimum parameter by military research authorities. We expect to tighten up the device parameters around this value for military use in the near future. Assays based on detection of differents level of injected paraoxons is presented in following table:

We can use the values in Table 2 as an orientation. Paraoxon was selected as a representative organophosphate, but no major differences are expected in the assay of other organophosphate pesticides or nerve agents. The obtained limits of detection for real time monitoring are approximately twenty times higher than the one when preincubation is used. On the other hand, the possibility of detecting organophosphates at picogram levels is sufficient for fast field analysis and continuous monitoring of endangered areas. The presented design is based on injection of a sample into the reaction mixture. In the future, we propose bleeding contaminated air through the reaction chamber so aerosol particles or contaminated air contaning organophosphates may be detected too. The scheme of a prospective implementation is shown in Figure 3.

The presented work represents a continuous effort to develop devices for military health defense purposes. Biosensors have been developed for characterization of antibodies [22, 23, 24] and several microorganisms [25] such as the biological agent *Francisella tularensis* [26] or model *Escherichia coli* [27] in real time; similar biosensors could even be applied for aflatoxin assays [28]. The last mentioned could act as an interfering compound; however, these compounds are quite hydrophobic and less likely to be found than organophosphates. We view the development of biosensors able to detect biological or chemical agents as a big challenge and we hope for a practical impact of our continued research.

## 3. Experimental Section

### 3.1 Enzymes and chemicals

Available acetylcholinesterases were compared and the human recombinant one was chosen as the best, due to its higher activity per milligram of protein. AChE (2,000 U/mg), together with acetylthiocholine chloride (ATChCl), gelatin, human serum albumin (HSA) and glutaraldehyde were obtained from Sigma-Aldrich (Czech Republic). Paraoxon-ethyl was purchased from Labor Dr. Ehrenstorfer-Schafers (Augsburg, Germany) and diluted into deionized water to 1 – 10 – 100 – 1,000 – 10,000 ppb. Deionized water was prepared with a MilliQ system (Millipore).

### 3.2 Device

An amperometric sensor strip (25×7 mm) was screen printed with three electrodes: a platinum working one, a Ag/AgCl reference one, and a platinum auxiliary. The amperometric strip, a connection unit and Bioanalyzer detector were obtained from BVT (Brno, Czech Republic). The detector was connected to a PC via a serial port and data were processed by the appropriate Bioanalyzer-BTA software (BVT, Brno, Czech Republic). The reaction chamber (polyethylene cylinder of 10 mm diameter) and a Teflon-coated stirrer were purchased from P-Lab (Prague, Czech Republic).

### 3.3 Immobilization procedure

The AChE immobilization procedure was optimized as summarized in the Results and Discussion part. In the first step, sample (1 μL) including 2 U of AChE was spread over the working electrode. After drying, the second layer (0.5 μL with 1 U of AchE) was added with one of four immobilization procedures: simple adsorption onto working electrode (no reagents for immobilization purposes), stabilization of AChE layer by vapor of glutaraldehyde in closed chamber with or without HSA (0.3 mg/mL; 0.5 μL), and capture of the AChE in gelatin (0.5 μL of 0.1% gelatin in water). Finally the sensor was washed with a gentle stream of deionized water.

### 3.4 Measuring protocol

The biosensor fixed into the connection unit was immersed in the reaction chamber with 1 mM ATChCl diluted in phosphate buffered saline (PBS), stirring was started and the equilibrium current of the non-inhibited biosensor *i_n_* was measured. After that, biosensor was removed from reaction chamber and immersed into a tube with sample for 15 min. Finally, the current after inhibition (*i_i_*) was measured in the reaction chamber. In another measuring setup, the current before inhbition (*i_n_)* was noted and the sample was injected into the reaction chamber (2 μL). The actual current after a predefined time was taken as the *i_i_* value. The chemical reactions behind the measurements are shown in the following equations:

$$\begin{array}{l} {\text{CH}}_{3}{\text{COSCH}}_{2}{\text{CH}}_{2}{\text{N}}^{+}{{(\text{CH}}_{3})}_{3}\overset{\text{AChE}/{\text{H}}_{2}\text{O}}{\underset{\text{paraoxon}}{⥇}}{\text{HSCH}}_{2}{\text{CH}}_{2}{\text{N}}^{+}{{(\text{CH}}_{3})}_{3}+{\text{CH}}_{3}{\text{COO}}^{-}+{\text{H}}^{+}\\ 2{\text{HSCH}}_{2}{\text{CH}}_{2}{\text{N}}^{+}{{(\text{CH}}_{3})}_{3}\overset{+410\text{mV}}{\underset{{-2\text{e}}^{-}}{⟶}}{{(\text{CH}}_{3})}_{3}{\text{N}}^{+}{\text{CH}}_{2}{\text{S}-\text{SCH}}_{2}{\text{CH}}_{2}{\text{N}}^{+}{{(\text{CH}}_{3})}_{3}+{2\text{H}}^{+} \end{array}$$

The first reaction catalyzed by immobilized AChE and inhibition by paraoxon is indicated by the cross. The second equation shows the oxidation of thiocholine at the working electrode [29].

### 3.5 Experimental data processing

The presented study includes two strategies for organophosphates assay. In the first variant, two values of current were measured: before inhibition (*i_n_*) and after sample application (inhibition) (*i_i_*). The most typical expression of output signals is by percent of inhibition *I*:

$$I=(1-\frac{i_{i}}{i_{n}})\times 100$$

In the second variant of the measurement process, the real time decrease of the detected current *i* is followed commonly by current noise. We recommend the simple symbols: “−” for no significant current decrease (S/N < 3), “+” for decrease of current at limit of detection level or higher (S/N≥3), and “++” for current decreases at the limit of quantification or higher (S/N≥10).

## Figures and Tables

**Table 1.** Summary of AChE immobilization procedures, maximal current provided by non-inhibited biosensor *i_n_* and percent of inhibition *I* are included. The dispersion indicates standard deviation.

**Immobilization procedure**	***i****_n_***(nA)**	***I*(%)**
Simple sorption	88±13	83.0
Glutaraldehyde cross-linking	109±17	86.2
Glutaraldehyde and HSA cross-linking	102±11	84.3
Capture in gelatin	160±19	90.6

**Table 2.** Performance of AChE based biosensor for real time assay of organophosphate paraoxon. The three symbols are used in this table as mark for the outputting value: − (no significant detection), + (decrease of current above limit of detection: S/N >3), ++ (decrease of current above limit of quantification S/N>10) when current measured one minute after paraoxon injection.

**paraoxon**	no (blank)	2 pg	20 pg	200 pg	2 ng	20 ng
**mark**	−	−	−	+	++	++

## References

[1] Kuca K., Jun D., Musilek K. (2006). Structural requirements of acetylcholinesterase reactivators. Mini Rev. Med. Chem..

[2] Kuca K., Cabal J., Kassa J. (2005). *In vitro* reactivation of sarin-inhibited brain acetylchoinesterase from different species by various oximes. J. Enz. Inhib. Med. Chem..

[3] Kuca K., Jun D., Bajgar J. (2007). Currently used cholinesterase reactivators against nerve agent intoxication: Comparison of their effectivity in vitro. Drug Chem. Toxicol..

[4] Pohanka M., Jun D., Kuca K. Photometric microplate assay for estimation of paraoxon inhibited acetylcholinesterase reactivation efficacy. J. Enz. Inhib. Med. Chem..

[5] Arduini F., Ricci F., Bourais L., Amine A., Moscone D., Palleschi G. (2005). Extraction and detection of pesticides by cholinesterase inhibition in a two-phase system: a strategy to avoid heavy metal interference. Anal. Lett..

[6] No H.Y., Kim Y.A., Lee Y.T., Lee H.S. (2007). Cholinesterase-based dipstick assay for the detection of organophosphate and carbamate pesticides. Anal. Chim. Acta.

[7] Kim B.M., Abd El-Aty A.M., Hwang T.E., Jin L.T., Kim Y.S., Shim J.H. (2007). Development of an acetylcholinesterase-based detection kit for the determination of organophosphorus and carbamate pesticide residues in agricultural samples. Bull. Kor. Chem. Soc..

[8] Skladal P. (1996). Biosensors based on cholinesterase for detection of pesticides. Food Technol. Biotechnol..

[9] Renault N.J. (2001). New trends in biosensors for organophosphorus pesticides. Sensors.

[10] Okazaki S., Nakagawa H., Fukuda K., Asakura S., Kiuchi H., Shigemori T., Takahashi S. (2000). Reactivation of an amperometric organophosphate pesticide biosensor by 2-pyridinealdoxime methochloride. Sens. Actuat. B.

[11] Longobardi F., Solfrizzo M., Compagnone D., Del Carlo M., Visconti A. (2005). Use of electrochemical biosensor and gas chromatography for determination of dichlorvos in wheat. J. Agric. Food Chem..

[12] Gulla K.C., Gouda M.D., Thakur M.S., Karanth N.G. (2002). Reactivation of immobilized acetyl choilnesterase in an amperometric biosensor for organophosphorus pesticide. Biochim. Biophys. Acta.

[13] Del Carlo M., Pepe A., De Gregorio M., Mascini M., Marty J.L., Fournier D., Visconti A., Compagnone D. (2006). An electrochemical bioassay for dichlorvos analysis in durum wheat samples. J. Food Prot..

[14] Skládal P., Krejčí J. (1996). Performance of the amperometricbiosensor with immobilized butyrylcholinesterase in organic solvents. Collect. Czech Chem. Commun..

[15] Pohanka M., Jun D., Kuca K. (2007). Amperometric biosensor for evaluation of competitive cholinesterase inhibition by the reactivator HI-6. Anal. Lett..

[16] Ciucu A., Ciucu C. (2002). Organic phase amperometric biosensor for detection of pesticides. Roum. Biotechnol. Lett..

[17] Suprun E., Evtugyn G., Budnikov H., Ricci F., Moscone D., Palleschi G. (2005). Acetylchoinesterase sensor based on screen-printed carbon electrode modified with prussian blue. Anal. Bioanal. Chem..

[18] Sotiropoulou S., Chaniotakis N.A. (2005). Lowering the detection limit of the acetylcholinesterase biosensor using a nanoporous carbon matrix. Anal. Chim. Acta.

[19] Timur S., Telefoncu A. (2004). Actylcholinesterase (AChE) electrodes based on gelatin and chitosan matrices for the pesticide detection. Artif. Cells Blood Substit. Immobil. Biotechnol..

[20] Andreescu S., Marty J.L. (2006). Twenty years research in cholinesterase biosensors: from basic research to practical applications. Biomol. Eng..

[21] Pohanka M., Kuca K., Jun D. (2007). Amperometric biosensor for pesticide methamidophos assay. Acta Medica.

[22] Pohanka M., Treml F., Hubálek M., Banďouchová H., Beklová M., Pikula J. (2007). Piezoelectric biosensor for a simple serological diagnosis of tularemia in infected european brown hares (*Lepus europaeus*). Sensors.

[23] Pohanka M., Pavlis O., Skládal P. (2007). Rapid characterization of monoclonal antibodies using the piezoelectric immunosensor. Sensors.

[24] Pohanka M., Pavliš O., Skládal P. (2007). Diagnosis of tularemia using piezoelectric biosensor technology. Talanta.

[25] Vitecek J., Petrlova J., Adam V., Havel L., Kramer K.J., Babula P., Kizek R. (2007). A fluorimetric sensor for detection of one living cell. Sensors.

[26] Pohanka M., Skládal P. (2007). Piezoelectric immunosensor for the direct and rapid detection of *Francisella tularensis*. Folia Microbiol..

[27] Pohanka M., Skládal P., Pavliš O. (2008). A label free piezoelectric immunosensor for rapid assay of *Escherichia coli*. J. Immunoass. Immunochem..

[28] Pohanka M., Kuca K., Jun D. (2008). Aflatoxin assay using an amperometric sensor strip and acetylcholinesterase as recognition element. Sens. Lett..

[29] Pohanka M., Jun D., Kalasz H., Kuca K. (2008). Cholinesterase biosensor construction – a review. Protein Peptide Lett..

